# A new species of *Falsopodabrus* Pic characterized with geometric morphometrics (Coleoptera, Cantharidae)

**DOI:** 10.3897/zookeys.614.6156

**Published:** 2016-09-01

**Authors:** Limei Li, Yaqing Qi, Yuxia Yang, Ming Bai

**Affiliations:** 1 The Key Laboratory of Zoological Systematics and Application, College of Life Sciences, Hebei University, Baoding 071002, Hebei Province, China; 2 Key Laboratory of Zoological Systematics and Evolution, Institute of Zoology, Chinese Academy of Sciences, Beijing 100101, China

**Keywords:** China, Falsopodabrus, geometric morphometrics, new faunistic record, new species, taxonomy

## Abstract

A new species of *Falsopodabrus* Pic, 1927 is described, *Falsopodabrus tridentatus* Yang, **sp. n.** (Yunnan, China). Geometric morphometric analyses based on the shapes of pronotum and hind wing and comparisons with two sibling species, *Falsopodabrus himalaicus* Wittmer, 1974 and *Falsopodabrus martensi* (Wittmer, 1979), support the valid status of the new species, also confirmed by the characters of tarsal claws. In addition to *Falsopodabrus himalaicus* and *Falsopodabrus martensi*, *Falsopodabrus kostali* Švihla, 2004 and *Falsopodabrus rolciki* Švihla, 2004 are recorded from China for the first time.

## Introduction

The genus *Falsopodabrus* was proposed by Pic (1927) for *Podabrus refossicollis* Pic, 1907, by monotypic and original designation. In this genus, *Falsopodabrus himalaicus* Wittmer, 1974 and *Falsopodabrus martensi* (Wittmer, 1979) are very similar, and they differ from each other only in the basal teeth of the tarsal claws of the anterior two pairs of legs, which are larger in the former species (Švihla 2004). During this study, another species from Yunnan, *Falsopodabrus tridentatus* sp. n., was found to share common characteristics with the two described species, and can be differentiated by the presence of basal teeth on all outer tarsal claws. Except for the differences in the claws, it was difficult to distinguish these species only by description or measurements, so Švihla (2004) suggested that additional research would be required to determine whether they are closely related species, subspecies, or merely a single species with variation along a cline.

In order to clarify the species complex of *Falsopodabrus himalaicus*, *Falsopodabrus martensi*, and *Falsopodabrus tridentatus* sp. n., the geometric morphometric technique is introduced in the present study. Geometric morphometrics offer a more comprehensive and effective approach to the study of shape through the multivariate statistical analysis of anatomical landmarks or outline of biological homology (Bookstein 1991; Rohlf and Marcus 1993; Adam et al. 2004). It preserves the information about the relative spatial arrangement of the data through the analysis (Zelditch et al. 2004), making it possible to find and analyze shape variations in the organisms within and between populations (Walker 2000). Moreover, geometric morphometric tools present the advantage of laying results that not only have high statistical power but also have easily visualized results, helping with their interpretation and communication (Rohlf and Marcus 1993; Adam et al. 2004; Zelditch et al. 2004). It has been successfully used to resolve taxonomic uncertainties and in delineating cryptic species of several beetle groups (i.e. Faille et al. 2008; Taravati et al. 2009; Hájek and Fikáček 2010; Xu et al. 2013), especially, it was shown to be a useful tool in discrimination of the cantharid species by analyzing the hind wing shape (Su et al. 2015).

In this article, except the hind wing, the shapes of aedeagus and abdominal sternite VIII of female which are in usual description of cantharid species are analyzed. The pronotum is traditionally measured by the ratio of length and width but fails to capture the geometrical relations between the anatomical points analyzed (Rohlf 1990), so it is also included in the analysis. The subjective of the study is to assess if *Falsopodabrus himalaicus*, *Falsopodabrus martensi*, and *Falsopodabrus tridentatus* sp. n. are separate species or conspecific, by using a geometric morphometric approach.

## Material and methods

The material is deposited in the following collections and the primary types were returned to the collections from which they were borrowed or were otherwise deposited in public museums.

CAS
California Academy of Sciences, San Francisco, USA 


IZAS
Institute of Zoology, Chinese Academy of Sciences, Beijing, China 


MHBU
Museum of Hebei University, Baoding, China 


MNHN
Muséum National d’Histoire Naturelle, Paris, France 


NHMB
 Naturhistorisches Museum Basel, Switzerland 


NMPC
 Narodni muzeum, Praha, Czech Republic 


The description format and the method used in this study follow that of Okushima and Yang (2013) and Yang et al. (2014). Morphological terminology of female genitalia follows that of Brancucci (1980) and hind wing of Kukalová-Peck and Lawrence (1993).

**Figure 1.**
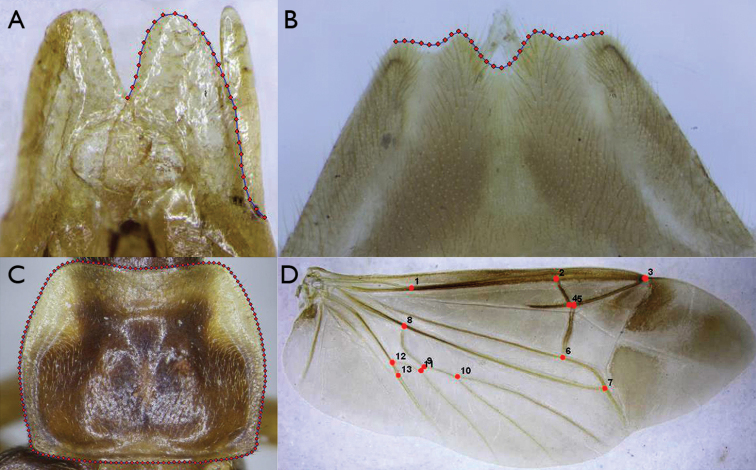
**A** Aedeagus **B** abdominal sternite VIII of female **C** pronotum **D** hind wing showing digitizing landmarks or points around the outline. **A–B** *Falsopodabrus himalaicus* **C–D** *Falsopodabrus tridentateus* sp. n.

Four morphological structures were analyzed by the geometric morphometrics, including pronotum, hind wing, aedeagus, and abdominal sternite VIII of female. The numbers of specimens studied for each structure of each species are indicated in the Table 1. All images were taken using a Canon 450D camera mounted on a Nikon SMZ1500 stereomicroscope, and were annotated using the TpsUtil software (Rohlf 2010a). The TpsDig2.16 software (Rohlf 2010b) was used to digitized outlines around the dorsal plate of the paramere of aedeagus (30 semi-landmarks), posterior margin of abdominal sternite VIII of female (30 semi-landmarks) and all margins of pronotum (150 semi-landmarks), and 13 landmarks at vein junctions of the hind wing as that of Su et al. (2015) (Fig. 1).

**Table 1.** The number of specimens of each species examined for each character used in the geometric morphometric analyses.

species	aedeagus	female abdominal sternite VIII	pronotum	hind wing
*Falsopodabrus himalaicus* Wittmer, 1974	9	11	39	28
*Falsopodabrus tridentatus* sp. n.	12	10	34	20
*Falsopodabrus martensi* (Wittmer, 1979)	3	9	12	12

The shapes of each structure among taxa were analyzed using MorphoJ software (Klingenberg 2011). The relative similarity and discrimination of the three species was analyzed using Canonical Variates Analysis (CVA). CVA is presented using the first two canonical axes. CVA finds shape values that maximize group means relative to variation within groups, by assuming that covariate matrices are identical (Klingenberg 2010). This is an effective method for detecting differences among taxa. The statistical significance of pairwise differences in mean shapes is determined using permutation tests (10 000 replications) with Procrustes and Mahalanobis distances. Both tests are used to assess significance because *P*-values can differ due to the anisotropy (direction dependency) of shape variation (Klingenberg and Monteiro 2005). The variability in the shape space was assessed using a Principal Component Analysis (PCA). To better visualize the shape variation, we presented the mean configuration of the analyzed structures for each species. The thin plate spline visualization (deformation grids) are used to portray the resulting shape variations. The goal of those morphometrical studies is to investigate the amount and the type of differences between populations. Since all analyses are performed with or without size provided similar results, those presented in this article deal only with shape.

## Results

**Figure 2.**
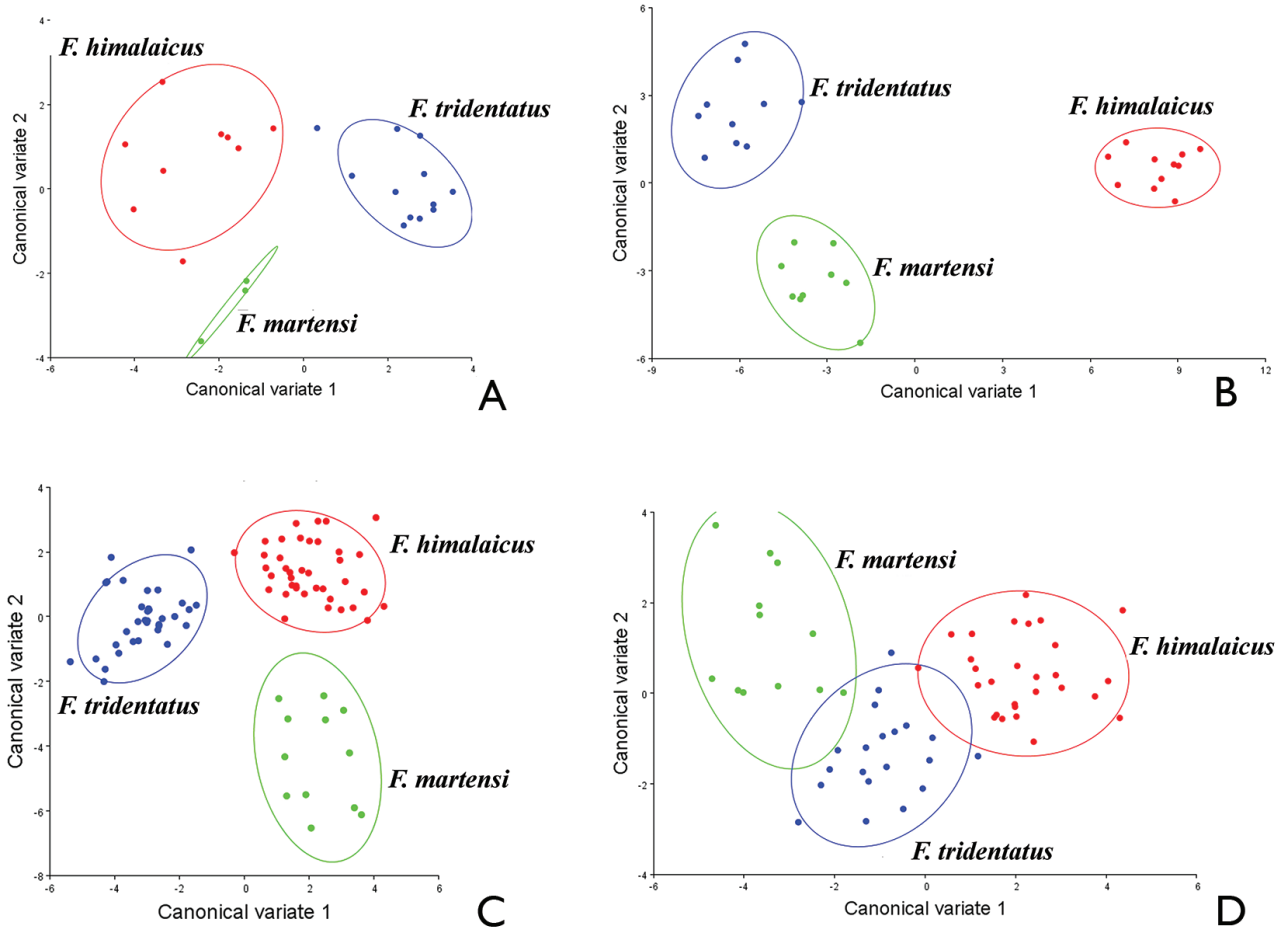
Plots of the first two canonical axes of Canonical Variates Analysis for *Falsopodabrus himalaicus*, *Falsopodabrus tridentatus* sp. n., and *Falsopodabrus martensi*, showing 90% confidence ellipses of population means: **A** aedeagus **B** abdominal sternite VIII of female **C** pronotum **D** hind wing.

The results provided by the CVs (Fig. 2) of shape differences for aedeagus, abdominal sternite VIII of female, pronotum, and hind wing all showed that *Falsopodabrus martensi*, *Falsopodabrus himalaicus* and *Falsopodabrus tridentatus* sp. n. occupied different areas of each graph respectively. Mahalanobis distances (*P* < 0.05) between the three species were highly significant in all pairwise comparisons, and Procrustes distances (*P* < 0.05) were similar (Tables 2, 3). However, measurements of the aedeagi were insignificantly different between *Falsopodabrus martensi* and *Falsopodabrus himalaicus* (Procrustes distances = 0.0524, *P* = 0.115) or *Falsopodabrus tridentatus* sp. n. (Procrustes distances = 0.0653, *P* = 0.1764), also difference was insignificant for the abdominal sternite VIII of females between *Falsopodabrus tridentatus* sp. n. and *Falsopodabrus martensi* (Procrustes distances = 0.0354, *P* = 0.0778). This suggested that the three species could be successfully delineated by the pronotum and hind wing, but were not fully sorted by the aedeagus and abdominal sternite VIII of female.

**Table 2.** Difference in shapes of aedeagus (left) and abdominal sternite VIII of female (right) among *Falsopodabrus himalaicus*, *Falsopodabrus tridentatus* sp. n., and *Falsopodabrus martensi*. Mahalanobis and Procrustes distances computed from a canonical variates analysis. *P*-values for the significance of the inter-population distances were computed using permutation tests (10 000 replications).

	*Falsopodabrus himalaicus*	*Falsopodabrus tridentatus*	*Falsopodabrus martensi*	*Falsopodabrus himalaicus*	*Falsopodabrus tridentatus*	*Falsopodabrus martensi*
Mahalanobis distances: *P*-values (above); distances between population (below)						
*Falsopodabrus himalaicus*	—	<.0001	0.0168	—	<.0001	<.0001
*Falsopodabrus tridentatus*	5.0884	—	<.0001	14.5081	—	<.0001
*Falsopodabrus martensi*	3.6005	5.0217	—	12.3302	6.477	—
Procrustes distances: *P*-values (above); distances between population (below)						
*Falsopodabrus himalaicus*	—	0.0024	0.115	—	<.0001	<.0001
*Falsopodabrus tridentatus*	0.0916	—	0.1764	0.1407	—	0.0778
*Falsopodabrus martensi*	0.0524	0.0653	—	0.1234	0.0354	—

**Table 3.** Difference in shapes of pronotum (left) and hind wing (right) among *Falsopodabrus himalaicus*, *Falsopodabrus tridentatus* sp. n., and *Falsopodabrus martensi*. Mahalanobis and Procrustes distances computed from a canonical variates analysis. *P*-values for the significance of the inter-population distances were computed using permutation tests (10 000 replications).

	*Falsopodabrus himalaicus*	*Falsopodabrus tridentatus*	*Falsopodabrus martensi*	*Falsopodabrus himalaicus*	*Falsopodabrus tridentatus*	*Falsopodabrus martensi*
Mahalanobis distances: *P*-values (above); distances between population (below)						
*Falsopodabrus himalaicus*	—	<.0001	<.0001	—	<.0001	<.0001
*Falsopodabrus tridentatus*	5.3921	—	<.0001	3.6132	—	<.0001
*Falsopodabrus martensi*	5.7685	6.9129	—	5.6757	3.6315	—
Procrustes distances: *P*-values (above); distances between population (below)						
*Falsopodabrus himalaicus*	—	<.0001	0.0444	—	0.001	0.0002
*Falsopodabrus tridentatus*	0.0206	—	<.0001	0.0221	—	0.001
*Falsopodabrus martensi*	0.0125	0.0300	—	0.0348	0.0303	—

**Figure 3.**
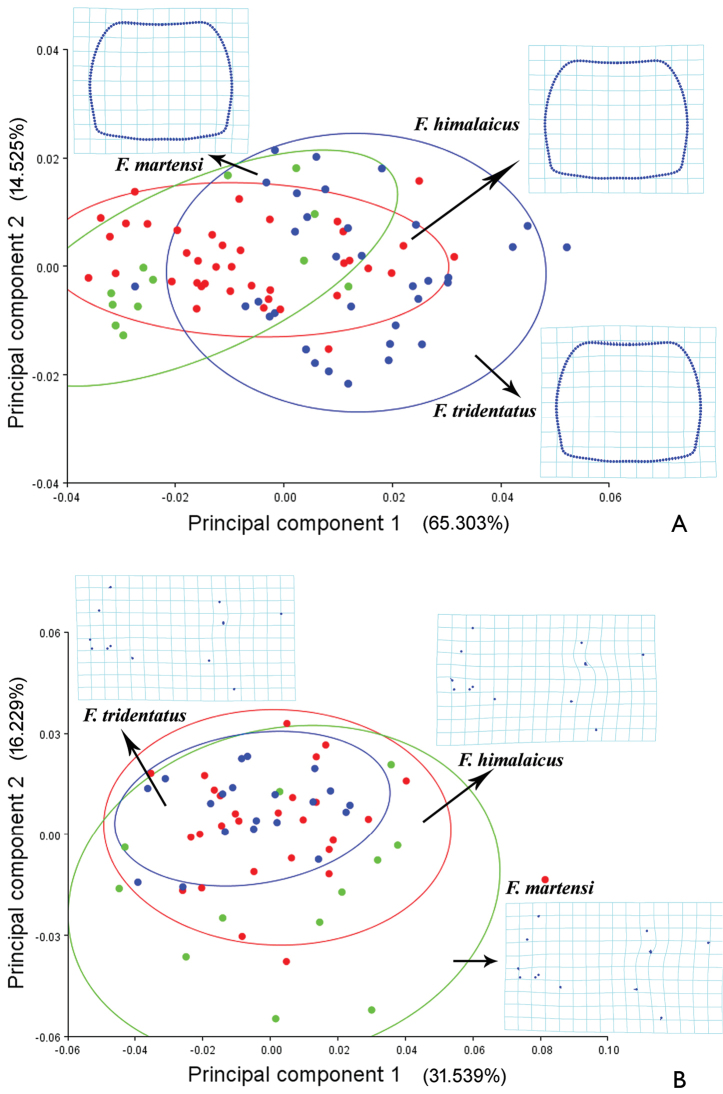
Plots of the first two components of Principal Component Analysis for *Falsopodabrus himalaicus*, *Falsopodabrus tridentatus* sp. n., and *Falsopodabrus martensi*, showing 90% confidence ellipses of population means: **A** pronotum **B** hind wing. The averaged shape of each species is depicted as deformations using thin plate splines.

To examine the differences of the pronotum and hind wing among *Falsopodabrus himalaicus*, *Falsopodabrus martensi*, and *Falsopodabrus tridentatus* sp. n., the shape variation for these structures are presented by the first two principal components of PCs (Fig. 3). The thin plate spline visualization (Fig. 3A) showed that the pronotum widened in *Falsopodabrus martensi*, while narrowed in *Falsopodabrus tridentataus* sp. n., presenting with anterior and posterior margins curved inwards while lateral margins curved outwards in the former, conversely in the latter species. For the hind wing (Fig. 3B), the radial cell (around by landmarks 2–5) is distinctly lengthened longitudinally in *Falsopodabrus himalaicus*, while shortened in the other two species, of which slightly in *Falsopodabrus tridentatus* sp. n. and distinctly in *Falsopodabrus martensi*; the distances between the vein junctions of RP & MP_1+2_ (landmark 7) and MP_1+2_ & MP_3+4_ (landmark 8) and between CuA_1_ & CuA_2_ (landmark 11) and CuA & CuA_1+2_ (landmark 12) both longest in *Falsopodabrus himalaicus*, while shortest in *Falsopodabrus martensi*; the angle formed by RA_3+4_ & r4 (landmark 5), r4 & RP (landmark 6) and RP & MP_1+2_ (landmark 7) widened in *Falsopodabrus himalaicus*, while narrowed in *Falsopodabrus martensi*.

The evidence above, shown by the significant shape differences in pronotum and hind wing, except the characteristic claws, suggest that *Falsopodabrus himalaicus*, *Falsopodabrus martensi*, and *Falsopodabrus tridentatus* sp. n. are separate species.

## Taxonomy

### Falsopodabrus tridentatus

Taxon classificationAnimaliaColeopteraCantharidae

Yang sp. n.

http://zoobank.org/444B1588-9D1D-4923-B0A2-A003D2A51544

Figs 4 , 5

#### Type material.

Holotype: ♂ (IZAS): China: Yunnan: Gaoligong Shan, Nujiang pref., 16.3 km W Gongshan, 2775m, 27.715°N, 98.502°E, 15.–19.VII.2000, H.-M. Gan, C. Griswold, D. Kavanaugh, H.-B. Liang, D. Ubick, D.-Z. Dong. Paratypes: China: Yunnan: 14♂, 2♀ (CAS): same data to holotype; 1♂, 2♀ (CAS): Gaoligong Shan, Nujiang Prefecture, Nujiang State Nature Reserve, No.12 Bridge Camp area, 16.3 km W of Gongshan, N27.71503° / E98.50244°, 2775m, 15.–19.VII.2000, Stop#00-23, D.H. Kavanaugh, C.E. Griswold, Liang H.-B., D. Ubick & Dong D.-Z. collectors; 1♀ (CAS): Gaoligong Shan, Nujiang Prefecture, Gongshan County, Danzhu He drainage, 13.5 air km SSW of Gongshan, 2700m, N27.63063° / E98.62074°, 30.VI–5.VII.2000, Stop#00-17, D.H. Kavanaugh, C.E. Griswold, Liang H.-B., D. Ubick, & Dong D.-Z. collectors; 1♀ (CAS): Fugong Couny, Lumadeng Township, Lao Shibali Yakou, 3270m, N27.06429° / E098.75123°, 13.VIII.2005, Stop#DNK- 2005-079, D.H. Kavanaugh, H.B. Liang, D.Z. Dong & G. Tang collectors; 1♀ (CAS): Fugong Couny, Lumadeng Township, Shibali area, 2535m, N27.16536° / E098.78003°, 4.–17.VIII.2005, Stop#DNK-2005-059, D.H. Kavanaugh, H.B. Liang, P. Paquin & D.Z. Dong, collectors; 1♂ (CAS): Fugong Couny, Lishadi Township, 10km W of Shibali on Shibali Road, 3221m, N27.20055° / E098.71399°, 5.–16.VIII.2005, pitfall traps, Stop#DHK-2005-061, D.H. Kavanaugh, P. Paquin & H.B. Liang collectors; 2♂, 3♀ (IZAS): Gongshan County, Qiqi Reserve, N27.43, E98.34, 2000m, 9.VII.2000, Sino-America Exped., Liang H.B; 1♀ (IZAS): Gongshan County, No. 12 Bridge, 2750m, N27.72, E98.60, 15.VII.2000, Sino-America Exped., Liang H.B.; 1♀ (IZAS): same data, 16.VII.2000; 1♂, 2♀ (IZAS): same data, 18.VII.2000.

#### Distribution.

China (Yunnan).

#### Etymology.

The specific name is derived from the Latin *tri* (three) and *dentatus* (tooth), referring to the presence of basal teeth on all outer claws.

#### Diagnosis.

The new species is similar to both *Falsopodabrus himalaicus* and *Falsopodabrus martensi*, but differs from the latter by the presence of basal teeth on all outer claws in both sexes.

#### Description.

**Figures 4.**
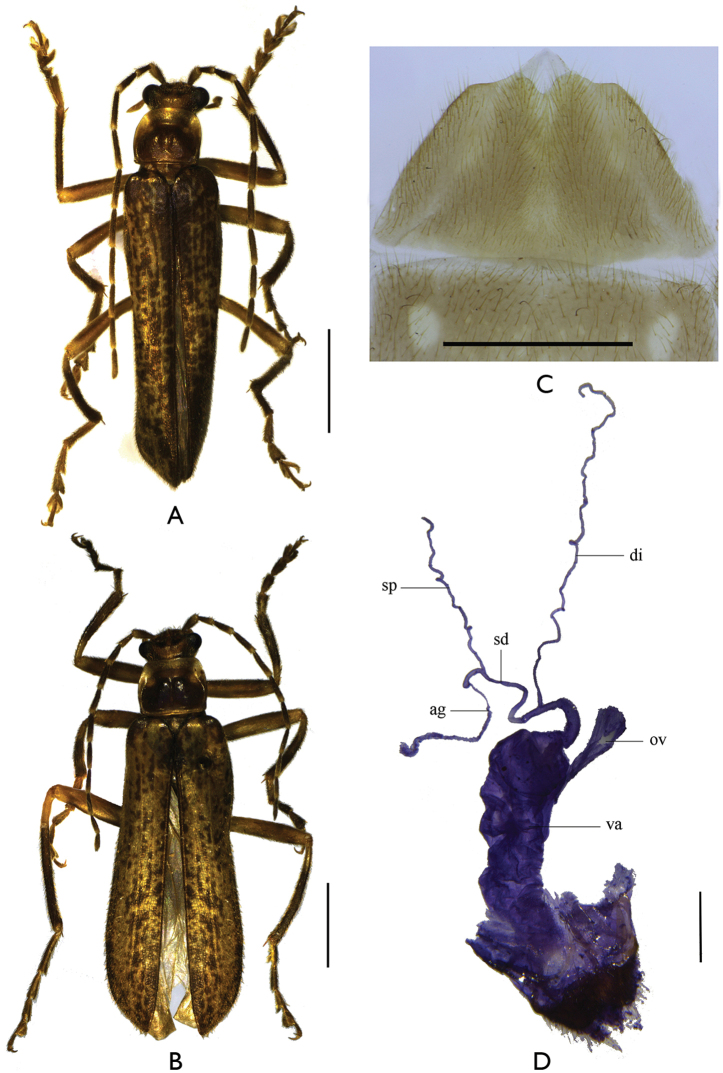
*Falsopodabrus tridentatus* sp. n. **A–B** habitus, dorsal view: **A** male **B** female **C** abdominal sternite VIII of female, ventral view **D** female genitalia, lateral view. The abbreviations: ag: accessory gland; di: diverticulum; sd: spermathecal duct; sp: spermatheca; ov: median oviduct; va: vagina. Scale bars **A–B**: 2.0 mm; **C–D**: 1.0 mm.

Male (Fig. 4A). *Head* black, pale brown on dorsum, each side with a dark brown marking behind antennal socket, mouthparts pale brown, maxillary and labial palpi darkened, apices of mandibles dark brown, antennae black, pale brown at apex of each antennomere, pronotum dark brown, more or less lightened at anterior part of disc, scutellum pale brown, elytra pale brown and mottled with irregular dark brown markings, legs pale brown, darkened at apices of femora and tarsomeres and bases of tibiae and coxae, more or less darkened at outer sides of femora and outer and dorsal sides of tibiae, prosternum pale brown, meso- and metasterna and abdominal ventrites black brown, pale brown at posterior margins of abdominal ventrites and the whole terminal ventrite. Body densely covered with short, recumbent, light yellow pubescence, mixed with slightly long, semi-erect, black brown setae on elytra.

*Head* with temples obliquely converging posteriorly, dorsum distinctly convex in central part, surface semilustrous, densely and finely punctate; eyes strongly protruding, head width across eyes distinctly wider than anterior margin of pronotum; terminal maxillary palpomeres long-triangular, widest at basal one-third, with apical parts of inner margins arcuate and sharp, acute at apices; antennae extending along basal two-thirds length of elytra, antennomeres II about three times as long as wide at apex, III about one-third longer than II, IV longest, IV–X each with a narrow, smooth, longitudinal impression nearly in middle of outer margin, which longest on V, XI pointed at apex.

*Pronotum* 1.1 times as wide as long, widest near middle, anterior margin slightly arcuate, lateral margins rounded, posterior margin arcuate and narrowly bordered, anterior angles rounded, posterior angles rectangular, disc convex on posterolateral parts, surface semilustrous, finely and sparsely punctate.

*Elytral* length about 5.5 times length of pronotum, 3.5 times as long as humeral width, with lateral margins nearly parallel, surface semilustrous, ruguse-lacunose and finely punctate.

**Figures 5.**
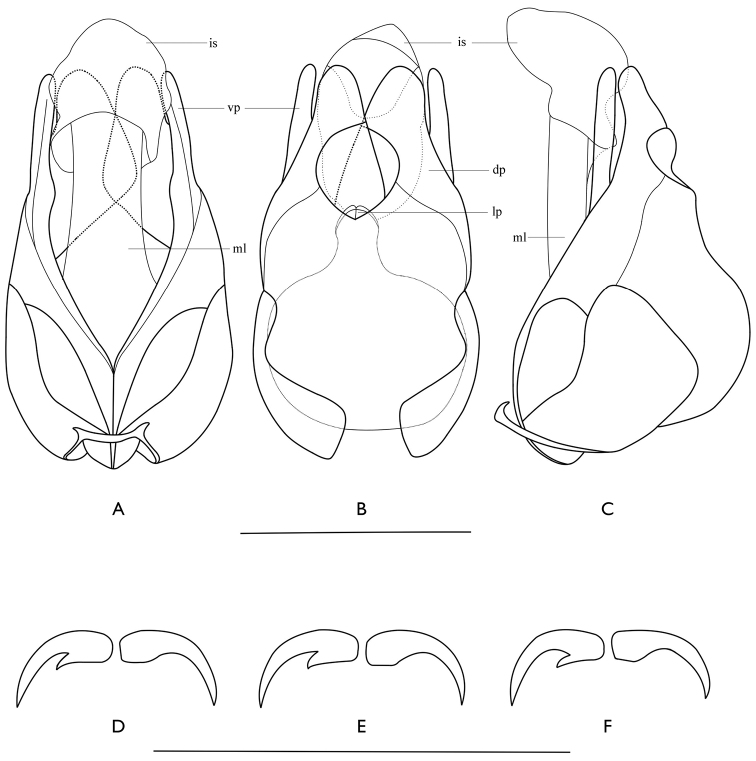
Male of *Falsopodabrus tridentatus* sp. n. **A–C** aedeagus (**A** ventral view B dorsal view **C** lateral view) **D–F** tarsal claws of left legs, dorsal view (**D** fore leg **E** middle leg **F** hind leg). The abbreviations: dp: dorsal plate of each paramere; is: inner sac of median lobe; lp: laterophyse; ml: median lobe; vp: ventral process of each paramere. Scale bars: 1.0 mm.

*Legs* with all outer tarsal claws each with a basal tooth (Fig. 5D–F).

*Aedeagus* (Fig. 5A–C): Inflated basally, ventral process of each paramere flattened and nearly straight, with apex rounded, dorsal plate almost as long as ventral process, rounded at apical margin, delaminated into two layers, ventral layer with inner margin largely triangularly protuberant in middle, dorsal layer with inner margin roundly emarginate at basal portion, laterophyse compressed, short and rounded at apex, leaning against each other and situated in middle of dorsal side of median lobe, inner sac of median lobe swollen and slightly lengthened ventrally, distinctly shorter than tegmen.

Female (Fig. 4B). Like male, except head less convex on dorsum, head width across eyes slightly wider than pronotum, antennae extending only to elytral midlength, antennomeres VI–X without impressions, pronotum 1.2 times wider than long, moderately convex on posterolateral parts of disc, elytra with lateral margins slightly diverging posteriorly, abdominal sternite VIII (Fig. 4C) evenly narrowed posteriorly, posterior margin moderately emarginate in middle and has rounded lobes on either side of middle emargination, nearly straight on lateral portions, present with a membranous triangular lobe behind the middle emargination. Internal reproductive organs (Fig. 4D): vagina elongate and abruptly extended apically as a thin and long duct; diverticulum and spermathecal duct arising from the end the long duct of vagina; diverticulum long, thin and spiral; spermathecal duct slightly thicker and distinctly shorter than diverticulum; spermatheca thin and spiral, distinctly shorter than diverticulum, basal portion extended into a very short tube, where accessory gland opening; accessory gland nearly as long as spermatheca; median oviduct attached near apex of vagina.

Variation within type series. Body length of the holotype: 9.0 mm, width: 1.7 mm; body length of male paratypes: 8.5–10.0 mm, width: 1.5–2.0 mm; body length of female paratypes: 9.0–11.0 mm, width: 1.7–2.2 mm.

#### Remarks.

Except the difference in the tarsal claws, the new species differs from its sibling species, *Falsopodabrus himalaicus* and *Falsopodabrus martensi*, also by the CVs which could be used for supporting evidence in confirming the species validity. The results of PCs show that the new species with pronotum is slightly narrower than the other two; hind wing with radial cell is least distorted, the distances are between the vein junctions of RP & MP_1+2_ and MP_1+2_ & MP_3+4_ and between CuA_1_ & CuA_2_ and CuA & CuA_1+2_ shorter than *Falsopodabrus himalaicus*, while longer than *Falsopodabrus martensi*, the angle formed by RA_3+4_ & r4, r4 & RP and RP & MP_1+2_ narrower than *Falsopodabrus himalaicus*, while wider than *Falsopodabrus martensi*.

### Falsopodabrus himalaicus

Taxon classificationAnimaliaColeopteraCantharidae

Wittmer, 1974

Falsopodabrus himalaicus Wittmer, 1974: 631, fig. 6.

#### Type material examined.

Holotype ♂ (NHMB): “Sikkim 11600' \ Yagtang”, “17.6.1959 \ F. Schmir”, “HOLOTYPUS”, “Falsopodabrus \ himalaicus \ Wittm. \ det. W. Wittmer”, “Naturhistorisches \ Museum Basel \ Coll. W. Wittmer”, “CANTHARIDAE \ CANTH00002544”.

#### Other material examined.

China: Xizang: 1♂, 9♀ (MHBU): Mainling, Zhaxiraodeng, 2.VIII.2008, leg. Z.J. Zhou; 1♂, 1♀ (MHBU): Mainling, Oglung, 14.VIII.2008, leg. Z.J. Zhou; 7♂, 7♀ (MHBU): Bomi, 3000 m, 20.VIII.2003, leg. G.D. Ren; 1♀ (MHBU): Bomi, 26.VII.2009, leg. G.D. Ren, Y.B. Ba & Z.J. Zhou; 3♀ (IZAS): Bomi, Yi’ong, 2700 m, 1.IX.1983, leg. Y.H. Han; 1♂, 2♀ (IZAS): Bomi, Yi’ong, 2300 m, 14.VIII.1983, leg. Y.H. Han; 1♂, 4♀ (IZAS): Bomi, Yi’ong, 2300 m, 25.VIII.1983, leg. Y.H. Han; 1♀ (IZAS): Mainling, 2950m, 19.VIII.1974, leg. F.S. Huang.

#### Distribution.

China (new country record: Xizang); India, Bhutan, Nepal (Kopetz, 2009).

### Falsopodabrus martensi

Taxon classificationAnimaliaColeopteraCantharidae

(Wittmer, 1979)

Stenothemus martensi Wittmer, 1979: 331.
Falsopodabrus martensi : Švihla 2004: 203, fig. 165.

#### Additional material examined.

China: Xizang: 1♂, 2♀ (IZAS): Mêdog, Tiqin, 3400 m, 7.IX.1982, leg. Y.H. Han; 1♂, 3♀ (IZAS): Mêdog, Nage, 3150 m, 22.VIII.1974, leg. F.S. Huang; 1♀ (IZAS): same data, 23.VIII.1974; 1♂, 2♀ (IZAS): Yadong, 2800 m, 23.VII.1960, leg. C.G. Wang; 1♀ (IZAS): Mêdog, 2750 m, 21.VIII.1983, leg. Y.H. Han.

#### Distribution.

China (new country record: Xizang); Nepal, India.

### Falsopodabrus kostali

Taxon classificationAnimaliaColeopteraCantharidae

Švihla, 2004

Falsopodabrus kostali Švihla, 2004: 204, figs 170–172, 216.

#### Type material examined.

Holotype ♂ (NMPC): “NE India; Meghalaya; 1400m; \ Nokrek N.P.; 3km S Daribokgiri; \ 25°27’N 90°19’E; 26.iv.1999; \ Koštäl Z. leg.”, “HOLOTYPUS \ Falsopodabrus \ kostali sp. n. \ V. Švihla det. 2003”.

#### Other material examined.

China: Xizang: 1♂ (IZAS): Xigonghu, 1450 m, 11.V.1983, leg. Y.H. Han; 1♀ (IZAS): Mêdog, Baibung, 850m, 17.V.1983, leg. Y.H. Han.

#### Distribution.

China (new country record: Xizang); India, Myanmar.

### Falsopodabrus rolciki

Taxon classificationAnimaliaColeopteraCantharidae

Švihla, 2004

Falsopodabrus rolciki Švihla, 2004: 203, figs 167–169, 215.

#### Type material examined.

Holotype ♂ (NMPC): “NE India; Meghalaya; 1999 \ 3km E of Tura; 500-1150m; \ 25°30’N 90°14’E; 1.-8.v. \ J. Rolčik leg.”, “HOLOTYPUS \ Falsopodabrus \ rolciki sp. n. \ V. Švihla det. 2003”.

#### Other material examined.

China: Xizang: 1♂ (NHMB): Tibet, Zayul, 12000ft, Summer 1937, R.I.H. Kaulback; 1♂ (IZAS): Mêdog, Baibung, 850m, 12.VI.1983, leg. Y.H. Han; 1♂ (IZAS): same data, 17.V.1983; 1♀ (IZAS): same data, 18.V.1983; 1♀ (IZAS): Mêdog, Xirang, 1150m, 7.VI.1983; 1♀ (IZAS): Mêdog, Didong, 1000m, 4.VI.1983, leg. Z. Lin; 2♂♂, 2♀♀ (MHBU): Zayü, Xia Zayü, 12.–13.VII.2005, leg. A.M. Shi.

#### Distribution.

China (new country record: Xizang); India, Myanmar.

### Falsopodabrus refossicollis

Taxon classificationAnimaliaColeopteraCantharidae

(Pic, 1907)

Podabrus refossicollis Pic, 1907: 175.
Stenothemus refossicollis Champion: 126. Synonymized by Wittmer 1974: 62.
Stenothemus championi Pic, 1927: 40 [replacement name for Stenothemus refossicollis Champion, 1926, nec Pic 1907].
Podabrus (Falsopodabrus) refossicollis : Pic 1927: 40.
Falsopodabrus refossicollis : Wittmer 1974: 62.

#### Type material examined.

Holotype ♂ (MNHN): “Kurseong” [India], “Podabrus \ refossicollis Pic“, “Falsopodabrus Pic“, “Falsopodabrus \ refossicollis \ Pic \ det. W. Wittmer”, “TYPE”.

#### Other material examined.

China: Xizang: 1♂ (IZAS): Zham, 2400 m, 4.VII.1975, leg. F.S. Huang; 1♀ (IZAS): Zham, 2200 m, 25.V.1975, leg. Z.Q. Wang; 1♂ (IZAS): Nyalam, Zham, 2200 m, 10.V.1966, leg. S.Y. Wang.

#### Distribution.

China (Xizang); India, Nepal (Okushima 1999).

## Discussion

Sibling species are expected to show high morphological similarity. However, some differences in morphology that allow discrimination can be found when morphometric approaches are used (Moraes et al., 2004). In the present study, the species complex of *Falsopodabrus himalaicus*, *Falsopodabrus tridentatus* sp. n., and *Falsopodabrus martensi* were successfully discriminated using CVA performed on the shape variables of pronotum and hind wing, but were not fully delineated by the aedeagus and abdominal sternite VIII of female.

The aedeagus is traditionally the most reliable method to identify the cantharid species, but male genital characters were not sufficient in delimiting the closely related species, as suggested by Barkalov and Ståhls (1997). Similarly, although female abdominal sternite VIII is considered useful in the species descriptions, such as *Falsopodabrus kostali* and *Falsopodabrus rolciki*, it seems to have little diagnostic value in delimitation here.

Surprisingly the shape of the pronotum shows high diagnostic value in delimiting these three sibling species. Also, the hind wing shape is again verified to be a good taxonomic character in discrimination of the cantharid species, as suggested by Su et al. (2015).

**Figure 6.**
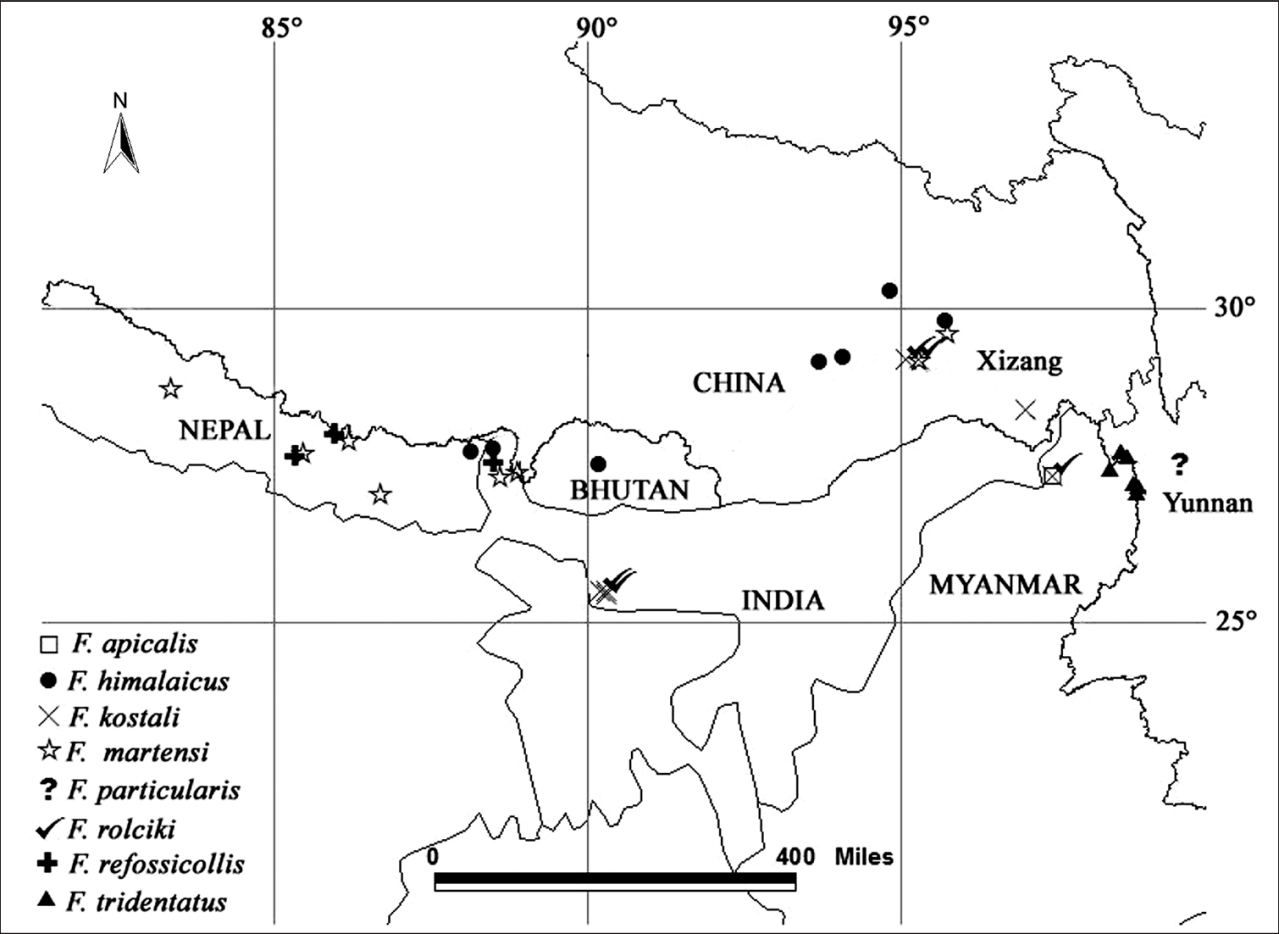
Distribution of *Falsopodabrus*. The location of *Falsopodabrus particularis* (Pic, 1931) lacks specific locality information in Yunnan Province, China.

Above all, the geometric morphometric results confirm the hypothesis proposed on the basis of tarsal claws morphology, so here we conclude that *Falsopodabrus himalaicus*, *Falsopodabrus tridentatus* sp. n., and *Falsopodabrus martensi* are morphologically similar but separate species. Now *Falsopodabrus* consists of eight species, which are all restricted to the Himalayan area (Fig. 6).

## Supplementary Material

XML Treatment for Falsopodabrus tridentatus

XML Treatment for Falsopodabrus himalaicus

XML Treatment for Falsopodabrus martensi

XML Treatment for Falsopodabrus kostali

XML Treatment for Falsopodabrus rolciki

XML Treatment for Falsopodabrus refossicollis
